# Comparing machine learning algorithms to predict 5-year survival in patients with chronic myeloid leukemia

**DOI:** 10.1186/s12911-022-01980-w

**Published:** 2022-09-06

**Authors:** Mostafa Shanbehzadeh, Mohammad Reza Afrash, Nader Mirani, Hadi Kazemi-Arpanahi

**Affiliations:** 1 Department of Health Information Technology, Faculty of Paramedical, Ilam University of Medical Sciences, Ilam, Iran; 2 Department of Health Information Technology and Management, School of Allied Medical Sciences, Shahid Beheshti University of Medical Sciences, Tehran, Iran; 3 Department of Treatment, Head of the Medical Truism, Zanjan University of Medical Sciences, Zanjan, Iran; 4 Department of Health Information Technology, Abadan University of Medical Sciences, Abadan, Iran; 5 Department of Student Research Committee, Abadan University of Medical Sciences, Abadan, Iran

**Keywords:** Machine learning, Data mining, Support vector machine, Leukemia, Survival

## Abstract

**Introduction:**

Chronic myeloid leukemia (CML) is a myeloproliferative disorder resulting from the translocation of chromosomes 19 and 22. CML includes 15–20% of all cases of leukemia. Although bone marrow transplant and, more recently, tyrosine kinase inhibitors (TKIs) as a first-line treatment have significantly prolonged survival in CML patients, accurate prediction using available patient-level factors can be challenging. We intended to predict 5-year survival among CML patients via eight machine learning (ML) algorithms and compare their performance.

**Methods:**

The data of 837 CML patients were retrospectively extracted and randomly split into training and test segments (70:30 ratio). The outcome variable was 5-year survival with potential values of alive or deceased. The dataset for the full features and important features selected by minimal redundancy maximal relevance (mRMR) feature selection were fed into eight ML techniques, including eXtreme gradient boosting (XGBoost), multilayer perceptron (MLP), pattern recognition network, k-nearest neighborhood (KNN), probabilistic neural network, support vector machine (SVM) (kernel = linear), SVM (kernel = RBF), and J-48. The scikit-learn library in Python was used to implement the models. Finally, the performance of the developed models was measured using some evaluation criteria with 95% confidence intervals (CI).

**Results:**

Spleen palpable, age, and unexplained hemorrhage were identified as the top three effective features affecting CML 5-year survival. The performance of ML models using the selected-features was superior to that of the full-features dataset. Among the eight ML algorithms, SVM (kernel = RBF) had the best performance in tenfold cross-validation with an accuracy of 85.7%, specificity of 85%, sensitivity of 86%, F-measure of 87%, kappa statistic of 86.1%, and area under the curve (AUC) of 85% for the selected-features. Using the full-features dataset yielded an accuracy of 69.7%, specificity of 69.1%, sensitivity of 71.3%, F-measure of 72%, kappa statistic of 75.2%, and AUC of 70.1%.

**Conclusions:**

Accurate prediction of the survival likelihood of CML patients can inform caregivers to promote patient prognostication and choose the best possible treatment path. While external validation is required, our developed models will offer customized treatment and may guide the prescription of personalized medicine for CML patients.

## Background

Leukemia is believed to be one of the most common and deadly known malignancies worldwide [1, 2]. It accounts for 4% of all malignancies and 4% of fatality rates emanating from malignancies [3]. Chronic myeloid leukemia (CML) is one of the most well-known forms of leukemia, accounting for 15 to 20% of all cases of leukemia. CML is a clonal myeloproliferative disorder (CMD) arising from acquired genetic alterations on the hematopoietic stem cells [4–6]. The chromosome abnormality known as the Philadelphia chromosome (Ph+) results from the fusion of the Abelson (Abl) tyrosine kinase gene at chromosome 9 and the breakpoint cluster (Bcr) gene at chromosome 22 (BCR-ABL fusion) [7, 8].

Today, there have been considerable advancements in leukemia management. Nonetheless, drug resistance, disease recurrence following treatment, cancer progression to advanced stages, disease prognosis, and survival prediction are of great significance [9]. Early detection of CML cases and active patient triaging help them evade the advanced stages of the disease and increase their survival chances [10, 11]. This requirement is more demanding since numerous clinical and non-clinical factors are involved in CML development [6, 10]. The decision about the best treatment path for an individual CML patient based on their specific clinical and demographic attributes and in the context of very effective treatment options is more multifaceted and often based on subjective evaluation. Furthermore, the existence of numerous disease severity levels and some uncertainty and ambiguities about the disease behavior and outcome further complicate the situation [12–15].

Conventional statistical methods provide forecasts without illuminating the meaning of the prediction or the associations amid numerous features that might influence patients’ survival. However, artificial intelligence (AI) technologies such as machine learning (ML) offers in-depth, effective, and non-invasive analytical capabilities over traditional statistical and experimental prediction methods in dealing with complex and ambiguous situations such as cancer outcome and survival prediction [16–24]. ML extracts comprehensible patterns and applied knowledge from large-scale raw datasets and thereby supports clinical decisions [25, 26].

So far, many studies have compared ML techniques for designing optimal and efficient clinical decision support systems (CDSSs) for the survival prognosis of patients with leukemia. Although a great number of studies have focused on acute myeloid leukemia (AML) [14, 25–30], CML [10, 31] has received little attention. Sasaki et al., suggested that identifying the most optimum and effective ML classifiers is necessary for improving therapeutic outcomes and increasing CML patients' life expectancy and survival [15].

Given the high incidence of CML in Iran and the lack of a reliable study to determine predictors of cancer survival using ML algorithms, the present study aimed to initially identify the most effective variables and feed them as data input into different ML techniques for a 5-year CML survival prognosis to assess their predictive power.

## Methods

### Study design

This was a retrospective and developmental study conducted in 2022 to predict 5-year survival in CML patients based on selected data-driven ML techniques.

### Study setting

**Fig. 1:**
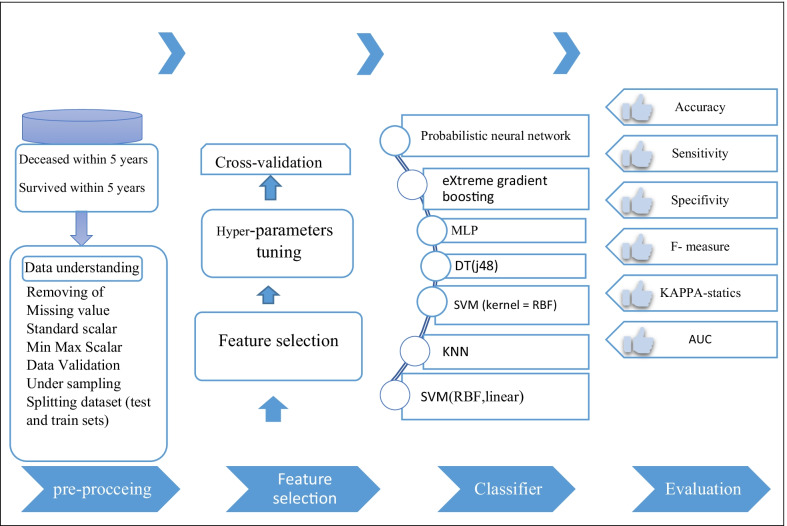
The roadmap of the proposed system based on the CRISP methodology. *SSVM* support vector machine, *RBF* radial basic function, *DT* decision tree, *KNN* k-nearest neighborhood, *XG Boost* eXtreme gradient boosting, *AUC* area under the curve

Our study was conducted in five main steps: data understanding, data preprocessing, feature selection, modeling, and evaluation. First, we aimed to recognize the most related variables to the 5-year CML survival prognosis and then use them as inputs for developing ML-based prediction models. To this end, we chose the most popular data mining method called cross-industry standard process (CRISP) to predict and diagnose CML. Figure 1 presents the proposed model of study steps based on the CRISP model. STATA and Python were used to provide descriptive statistics and data analysis. The scikit-learn library in Python was also used to implement the models.

### Data understanding

The data used in this study were obtained from a database at the Abadan University of Medical Sciences collected from April 2016 to December 2018. The primary dataset contained the information of 1218 patients with CML. The patients would be included in the study only if they met all the following criteria: (1) The patients were diagnosed with CML; (2) their survival status (alive/deceased) was available in their records; (3) in terms of the time frame, we considered patients diagnosed between 2011 and 2016 to have adequate follow-up period (5 years or more) after the diagnosis; (4) the age of more than or equal to 18 years; patients aged under 18 years old should be included in the scope of pediatric exploration [32]; (5) records with missing values of less than 30%. Accordingly, from 1218 patient records, 173 records for patients who were aged < 18 years old were excluded. In the preprocessing phase, 208 incomplete rows of data (with missing data of greater than 70%) were removed. After these criteria were applied, a total of 837 patient records were deemed suitable for inclusion (740: survived within 5 years and 97: deceased within 5 years).

### Study variables

Several variables were collected for CML patients in the EMR database. We checked the definition of the variables included in the data dictionary section of the database to completely understand the definitions of the data and the choice of proper variables. The criteria for selecting the candidate variables related to CML for survival prediction were based on consultations with expert oncologists and studying the relevant literature. Survival at 5 years and more was selected as the outcome variable. Survival is a continuous variable with units in months. Thus, we created a binary variable where any patient with a survival of 60 months or more was coded “yes”, or “no” otherwise. The following covariates were extracted based on the literature review coupled with experts’ opinions from the EMR database. Finally, a total of forty-five independent variables were utilized to predict the 5-years survival of patients with CML (dependent variable). These variables were categorized as demographic (two variables), history (seven variables), clinical manifestations (22 variables), and laboratory (14 variables) (see Table 1). After reviewing the patients’ variables, statistical analysis was performed to describe the differences in their features with the target variable (deceased within 5 years or survived). To this end, differences in demographic, history, clinical manifestations, and laboratory information of patients were described based on whether the patients with CML were deceased within 5 years or not, and the relationship of each feature with survival status was checked by the chi-square test.

**Table 1:** Baseline predictor variables

Data class	Types of variables	Variable	Range	Deceased within 5 years	Survived within 5 years	*p *value
				Total	Total	
Basic data	Independent Variables	Age (years)	18–45	19	121	0.081
			45–65	36	295	
			65–100	42	324	
		Gender	Male	65	480	0.093
			Female	32	260	
History		Radiation exposure	Yes–No	25–72	85–655	0.805
		Previous cancer treatment	Yes–No	22–57	36–704	0.692
		Genetic disorders	Yes–No	6–91	13–727	0.811
		Family history of leukemia	Yes–No	14–83	39–701	0.957
		Tobacco smoke	Yes–No	9–88	41–699	0.561
		Pesticides and industrial solvents	Yes–No	8–89	58–682	0.374
		Exposure to certain chemicals^a^	Yes–No	5–92	11–729	0.459
Manifestations		Fever	Yes–No	49–48	208–532	0.671
		Chill	Yes–No	17–80	148–592	0.759
		Swollen lymph nodes	Yes–No	36–61	108–632	0.714
		Petechiae	Yes–No	21–76	158–582	0.920
		Easy bleeding or bruising	Yes–No	26–71	128–612	0.802
		Recurrent nosebleeds	Yes–No	14–83	89–651	0.981
		Frequent or severe infections	Yes–No	36–61	280–460	0.059
		Arthralgia	Yes–No	29–68	211–485	0.630
		Headache	Yes–No	36–61	248–492	0.710
		Malaise	Yes–No	25–72	305–435	0.837
		Dyspnea	Yes–No	8–89	63–677	0.910
		Dizziness	Yes–No	6–91	58–682	0.891
		Visual disturbances	Yes–No	13–84	62–678	0.452
		Nausea/vomiting	Yes–No	88	119	0.100
		Ankle edema	Yes–No	64	132	0.924
		Weakness	Yes–No	51	79	0.130
		Sweats	Yes–No	102	140	0.092
		Weight loss	Yes–No	63–34	297–443	0.721
		Bone pain	Yes–No	12–85	165–575	0.816
		Spleen palpable	Yes–No	27–70	117–623	0.649
		Pain or a sense of "fullness" in the belly	Yes–No	22–75	86–654	0.930
		Feeling full after eating even a small amount of food	Yes–No	19–78	91–649	0.922
Laboratory		BCR-ABL (Philadelphia chromosome)	Positive–negative	88–9	82–658	0.631
		Anemia	Yes–No	43–54	215–489	0.052
		Poor appetite	Yes–No	37–60	119–621	0.760
		Areas of bone damage	Yes–No	10–87	74–666	0.058
		Increased leucocyte count	> 50 × 10^3^ ml	63	565	0.041
		Neutrophil proportion	> 72.6%	53	445	0.029
		Elevated blast cell proportion	> 10%	32	396	0.042
		Increased eosinophil count	> 0 /5 × 10^3^uL	66	321	0.049
		Increased basophil count	> 0 /1 × 10^3^uL	48	625	0.018
		Decreased platelet counts	< 150 × 10^3^ ml	29	108	0.052
		Increased neutrophil alkaline phosphatase	> 20 per 100 score neutrophils	52	256	0.049
		Resistance to tyrosine kinase inhibitors	Yes–No	24–73	268–472	0.072
Outcome variable	Dependent variable	Five-years survival statues	Deceased within 5 years/survived within 5 years	97	740	–

Exposure to certain chemicals, such as benzene—which is found in gasoline and is used by the chemical industry—is linked to an increased risk of some kinds of leukemia

### Pre-processing step

Data preprocessing is an essential step in the CRISP methodology to obtain an optimal, accurate, and beneficial dataset for further ML algorithms. In this study, many pre-processing methods on the dataset were applied before the training of the ML algorithms. Removal of missing values, standard scalar, Min–Max scalar, data validation, under-sampling, and splitting the dataset were examined to obtain an optimal dataset.

### Feature selection

Data mining algorithms usually have difficulty dealing with a large number of input variables, which poses a serious challenge to researchers. These irrelevant variables diminish the performance of many ML algorithms. Thus, the selection of important variables is a major step during data mining [33, 34]. Comparison of data mining results before and after feature selection in many studies has shown an improvement in performance criteria through feature selection. Similarly, in the present study, this approach (before and after) was adopted [35, 36]. Here, the minimal redundancy maximal relevance (mRMR) feature selection algorithm was employed. This technique uses a heuristic method to select the most relevant variables. The heuristic search that is utilized in the mRMR technique chooses optimum variables that have maximum relevance and minimum redundancy [37].

### Classification algorithms

To predict the survival chance of CML patients, eight ML techniques, including XGBoost, k-nearest neighborhood (KNN), pattern recognition network, probabilistic neural network, multilayer perceptron (MLP), support vector machine (SVM) (kernel = linear), SVM (kernel = RBF), and J-48 were employed. Although there are many supervised ML techniques, these particular models were chosen because they represent a range of modern and common methods in cancer research.

### Performance evaluation of classification algorithms

The k-fold cross-validation method was utilized to assess the performance of the examined data mining models and to compare the results of the classification models. Cross-validation is a resampling technique applied for the evaluation of data mining techniques in an unseen data sample. In this method, the ML models are trained and tested k times Additionally, to compare the performance of classification models, the mean of evaluation metrics such as accuracy, specificity, sensitivity, kappa, and area under the curve (AUC) were used (Eqs. 1–5).

1 $${\text{classification accuracy}} = \frac{{{\text{TP}} + {\text{TN}}}}{{{\mathrm{TP}} + {\text{TN}} + {\text{FP}} + {\text{FN}}}}{*}100$$

2 $${\text{classification sensitivity}} = \frac{{{\text{Tp}}}}{{{\mathrm{TP}} + {\text{FN}}}}{*}100$$

3 $${\text{classification specificity}} = \frac{{{\text{TN}}}}{{{\mathrm{TN}} + {\text{FP}}}}{*}100$$

4 $${\text{classification error}} = \frac{{{\text{FP}} + {\text{FN}}}}{{{\mathrm{TP}} + {\text{TN}} + {\text{FP}} + {\text{FN}}}}{*}100$$

5 $${\text{f-measure}} = 2{ }\frac{{\text{precision*sensitivity}}}{{{\mathrm{precision}} + {\text{sensitivity}}}}$$

### Ethical considerations

This study was approved by the Ethical Committee Board, Abadan University of Medical Sciences (code: IR.ABADANUMS.REC.1401.042). To protect the privacy of the patients and the confidentiality of the data, we concealed the unique identifying information of all the patients in the data collection process.

## Results

### Patient characteristics

Overall, 837 patients with CML met the predefined inclusion criteria. Of 837 eligible patients in our study, 545 (65.11%) were men and 292 (34.89%) were women, and the median age of the participants was 57.25 years (interquartile 18–100). Of these, 740 (88.41%) cases survived and 97 (11.58%) died. Table 1 provides a detailed description of all the variables.

The mean age of CML patients who died within 5 years was 60 ± 2 years old, and the mean age of the patients who survived within 5 years was 57 ± 1 years old (*p* value = 0.081). Table 1 shows that there was a significant association between some variables of patients who survived within 5 years or not: feature with *p* value < 0.005 which has a significant difference in patients who survived within 5 years or not. For example, the results showed that there was a significant relationship between elevated blast cell proportion and increased basophil count with the survival status of the patient with CML (*p* value = 0.042 and *p* value = 0.018, respectively) (see Table 1).

### Selection of patient features

**Fig. 2:**
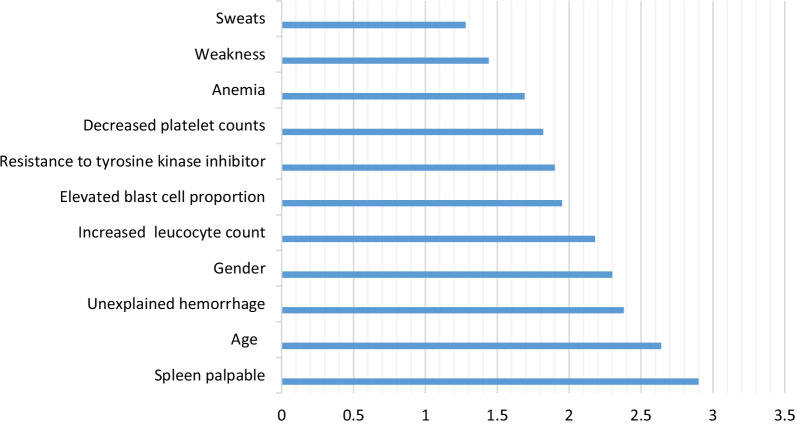
The most important variables selected by the mRMR

A set of 12 most important features to predict the 5-year survival of CML patients were selected based on a heuristic method. The selected features and their scores are ranked and represented in Fig. 2.

According to Fig. 2, spleen palpable, age, unexplained hemorrhage, sex, increased leucocyte count, elevated blast cell proportion, resistance to tyrosine kinase inhibitors, decreased platelet counts, anemia, malaise, and night sweats obtained the highest score for prediction of the 5-year CML survival.

### Results of hyperparameter tuning

To use ML classifiers more accurately and sensitively, the RandomizedSearchCV method was used for parameter tuning and optimization models, including XGBoost, MLP, KNN, probabilistic neural network, pattern recognition network, SVM (kernel = linear), SVM (kernel = RBF), and J-48 decision tree classifiers (see Table 2).

**Table 2:** Best hyperparameters of all the trained algorithms

Num	Data mining Models	Hyper-parameters	f-score
1	Decision tree (j48)		
2	MLP classifier	‘Learning rate’ = ’constant’, hidden_layer_size’ = (100,100,100), ‘alpha’ = 0.05, ‘activation’ = ’rulo’	87.6
3	SVM (kernel = linear)	C = 100, G = 0.0001	83.04
4	SVM (kernel = RBF)	C = 10, G = 0.001	81.9
5	XG Boost Classifier	‘min_chid_weigh’ = 1’max_depht’ = 12,’learning_rate’ = 0.1, ‘gamma’ = 0.4, ‘colsample_bytree’ = 0.3	81.02
6	KNN	K = 5	67.1
7	Pattern recognition network	57-10-5-2	69.02
8	Probabilistic neural network	57-2, Spread = 0.1	70.01

*SVM* support vector machine, *XG Boost* eXtreme gradient boosting, *KNN* K-nearest neighborhood

### Results of k-fold cross-validation for the performance of classification algorithms

The tenfold cross-validation method splits the dataset into 10 parts and performs the holdout method 10 times. The algorithms were run for both the full and the selected features of the dataset. Accordingly, the dataset was randomly divided into training (70%) and test parts (30%) for all algorithms. First, the algorithms were trained using the training section and then validated using the test section to determine predictions. The dataset for both full and selected features was examined using eight classification techniques. Firstly, we trained and tested the ML techniques of the full-features dataset, and the second time, we fed the selected features to the ML classifiers. To compare the performance of classification techniques with a 95% confidence interval (CI), the average assessment metrics were obtained. Table 3 presents the results of eight classification algorithms based on the selected features to predict 5-year CML survivability.

**Table 3:** Performance evaluation of the selected ML algorithms

Classifiers	MLP		KNN		DT (j48)		Pattern recognition network			XG Boost		Probabilistic neural network		SVM (kernel = RBF)		SVM (kernel = linear)	
	Full feature	Selected Feature	Full feature	Selected Feature	Full feature	Selected Feature	Full feature		Selected Feature	Full feature	Selected Feature	Full feature	Selected Feature	Full feature	Selected Feature	Full feature	Selected Feature
Mean Accuracy	0.67	0.77	0.62	0.68	0.73	0.83	0.62		0.68	0.69	0.79	0.62	0.69	0.69	0.85	0.69	0.83
95% confidence interval	(0.66, 0.68)	(0.76, 0.781)	(0.59, 0.66)	(0.671, 0.71)	(0.71, 0.75)	(0.834, 0.848)	(0.611, 0.64)		(0.68, 0.7)	(0.68, 0.7)	(0.77, 0.81)	(0.62, 0.63)	(0.691, 0.71)	(0.69, 0.71)	(0.82, 0.85)	(0.69, 0.71)	(0.82, 0.84)
Standard deviation	0.01	0.09	0.05	0.02	0.02	0.01	0.02		0.01	0.01	0.02	0.01	0.01	0.01	0.02	0.01	0.01
Mean Specificity	0.68	0.76	0.62	0.66	0.74	0.81	0.62		0.68	0.68	0.76	0.62	0.68	0.69	0.85	0.69	0.82
95% confidence interval	(0.67, 0.71)	(0.75, 0.77)	(0.58, 0.66)	(0.651, 0.71)	(0.731, 0.75)	(0.80, 0.82)	(0.61, 0.64)		(0.67, 0.691)	(0.67, 0.69)	(0.75, 0.77)	(0.62, 0.63)	(0.68, 0.7)	(0.68, 0.7)	(0.85, 0.86)	(0.68, 0.7)	(0.816, 0.83)
Standard deviation	0.02	0.01	0.07	0.02	0.01	0.09	0.02		0.01	0.01	0.01	0.01	0.01	0.01	0.01	0.01	0.01
Mean Sensitivity	0.71	0.72	0.62	0.70	0.74	0.83	0.61		0.70	0.70	0.78	0.62	0.71	0.71	0.86	0.70	0.83
95% confidence interval	(0.71, 0.73)	(0.71, 0.74)	(0.57, 0.68)	(0.68, 0.73)	(0.73, 0.752)	(0.83, 0.85)	(0.591, 0.64)		(0.69, 0.72)	(0.69, 0.72)	(0.78, 0.79)	(0.61, 0.65)	(0.7, 0.73)	(0.7, 0.73)	(0.86, 0.87)	(0.7, 0.72)	(0.82, 0.84)
Standard deviation	0.01	0.01	0.08	0.03	0.02	0.09	0.03		0.02	0.02	0.01	0.03	0.03	0.02	0.01	0.01	0.01
Mean area under the curve	0.70	0.76	0.62	0.69	0.75	0.83	0.62		0.69	0.69	0.76	0.62	0.70	0.70	86.1%	0.70	0.83
95% confidence interval	(0.69, 0.71)	(0.751, 0.774)	(0.610, 0.630)	(0.671, 0.712)	(0.731, 0.76)	(0.83, 0.85)	(0.61, 0.63)		(0.68, 0.7)	(0.68, 0.71)	(0.75, 0.778)	(0.62, 0.64)	(0.69, 0.71)	(0.69, 0.71)	(0.85, 0.86)	(0.69, 0.71)	(0.82, 0.84)
Standard deviation	0.01	0.09	0.01	0.02	0.01	0.01	0.01		0.01	0.01	0.01	0.01	0.01	0.01	0.01	0.014	0.01
Mean F1-score	0.70	0.76	0.61	0.68	0.73	0.83	0.62		0.69	0.69	0.77	0.62	0.70	0.72	0.87	0.69	0.82
95% confidence interval	(0.69, 0.71)	(0.751, 0.772)	(0.61, 0.63)	(0.671, 0.71)	(0.72, 0.74)	(0.83, 0.851)	(0.611, 0.63)		(0.68, 0.7)	(0.68, 0.71)	(0.76, 0.78)	(0.61, 0.64)	(0.69, 0.71)	(0.69, 0.71)	(0.86, 0.88)	(0.69, 0.71)	(0.821, 0.84)
Standard deviation	0.01	0.08	0.01	0.03	0.01	0.01	0.01		0.01	0.01	0.01	0.01	0.01	0.01	0.02	0.01	0.01
Kappa Statistic (KS)	0.7201	76.2%	0.612	0.681	0.701	83.2%	0.622		0.671	0.621	78.2%	0.602	0.718	0.752	0.861	0.681	0.831
	(0.71, 0.73)	(0.75, 0.771)	(0.61, 0.63)	(0.66, 0.69)	(0.70, 0.71)	(0.828, 0.85)	(0.59, 0.63)	(0.66, 0.69)		(0.61, 0.63)	(0.77, 0.79)	(0.58, 0.62)	(0.68, 0.73)	(0.74, 0.76)	(0.85, 0.86)	(0.67, 0.70)	(0.82, 0.84)
	0.01	0.01	0.01	0.02	0.00	0.08	0.07	0.01		0.02	0.01	0.05	0.04	0.01	0.01	0.01	0.01

*SVM* support vector machine, *RBF* radial basic function, *DT* decision tree, *KNN* k-nearest neighborhood, *XG Boost* eXtreme gradient boosting

Table 3 represents the performance of eight ML techniques on the selected feature in 10 independent run times. According to the results, the performance of ML models on the selected variables dataset was higher than that of the full-features dataset. When both the selected and full-features datasets were separately fed into the MLP model, the MLP classifier obtained a mean accuracy of 77%, sensitivity of 72%, specificity of 76%, F-measure of 76%, and kappa statistic of 76.2% on the selected features. It also obtained a mean accuracy of 69%, sensitivity of 71%, specificity of 68%, F-measure of 70%, kappa statistic of 72.2%, and AUC of 70% on the full-features dataset.

The J-48 model was applied to both the selected-features and the full-features data set. As shown in Table 3, by feeding the selected features into the J-48 algorithm, an average accuracy of 83%, a sensitivity of 83%, specificity of 81%, F-measure of 77%, kappa statistic of 83.2%, and AUC average of 83% were obtained. The results of J-48 on the full-features dataset were obtained as follows: an average accuracy of 69%, a sensitivity of 69%, specificity of 68%, F-measure of 69%, kappa statistic of 61%, and AUC average of 69%.

As shown in Table 3, when the selected dataset was used, the XGBoost classifier obtained an average accuracy of 79%, a sensitivity of 78%, specificity of 76%, F-measure of 77%, a kappa statistic of 78.2%, and an AUC average of 76%. The results of the XGBoost classifier on the full-features dataset indicated an average accuracy of 69.3%, a sensitivity of 70.6%, specificity of 68.4%, F-measure of 69.2%, a kappa statistic of 62.1%, and an AUC average of 69.5%.

**Fig. 3:**
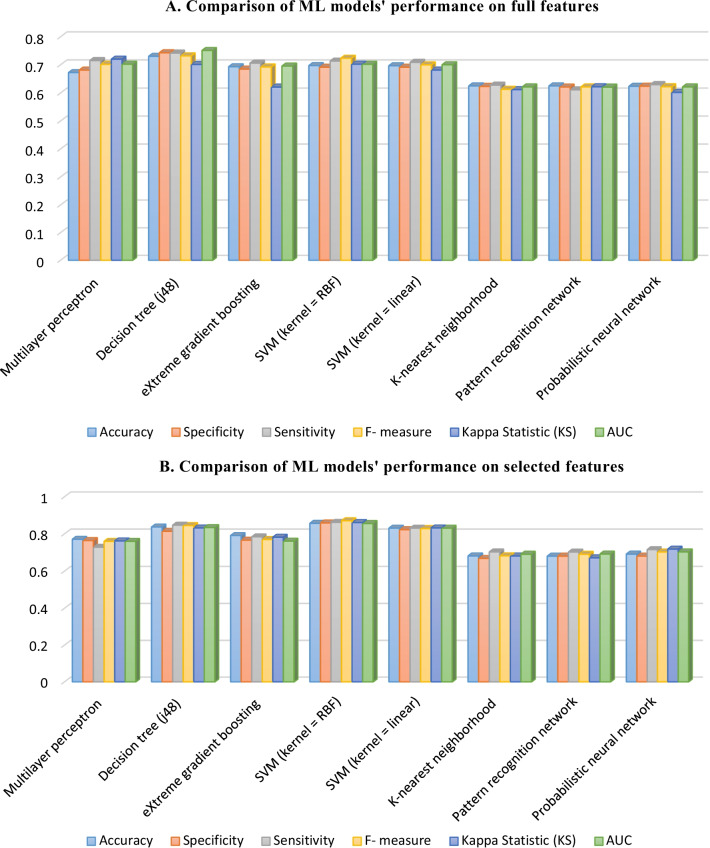
Comparison of ML models' performance on **A** full and **B** selected features

By using the selected-features dataset, the SVM (kernel = RBF) represents good results with an average accuracy of 85.7%, specificity of 85%, sensitivity of 86%, F-measure of 87%, kappa statistic of 86.1%, and AUC of 85%. Application of the SVM model to the full-features dataset also yielded an average accuracy of 69.7%, specificity of 69.1%, sensitivity of 71.3%, F-measure of 72%, kappa statistic of 75.2%, and AUC of 70.1%. The results of all experimented ML algorithms on the selected-features and full-features datasets are depicted in Fig. 3.

**Fig. 4:**
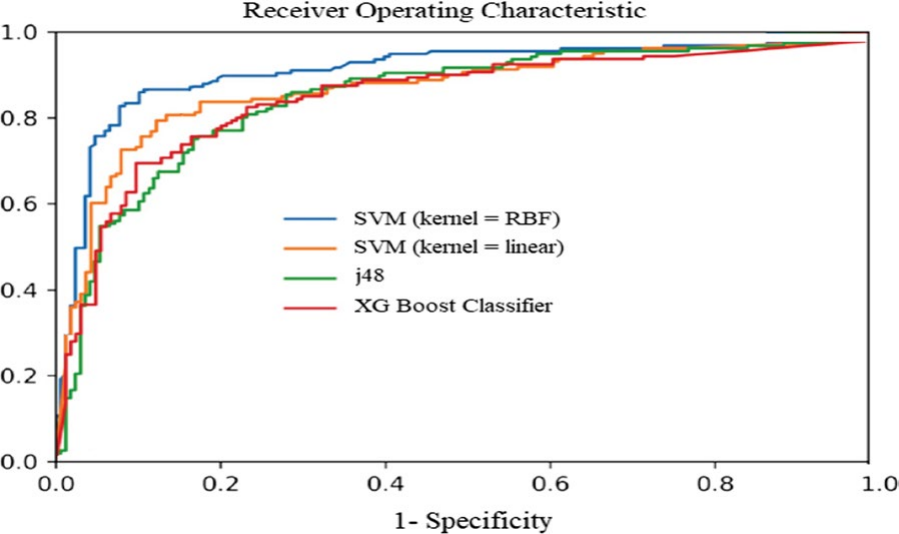
The ROC curve for the top four ML algorithms

**Fig. 5:**
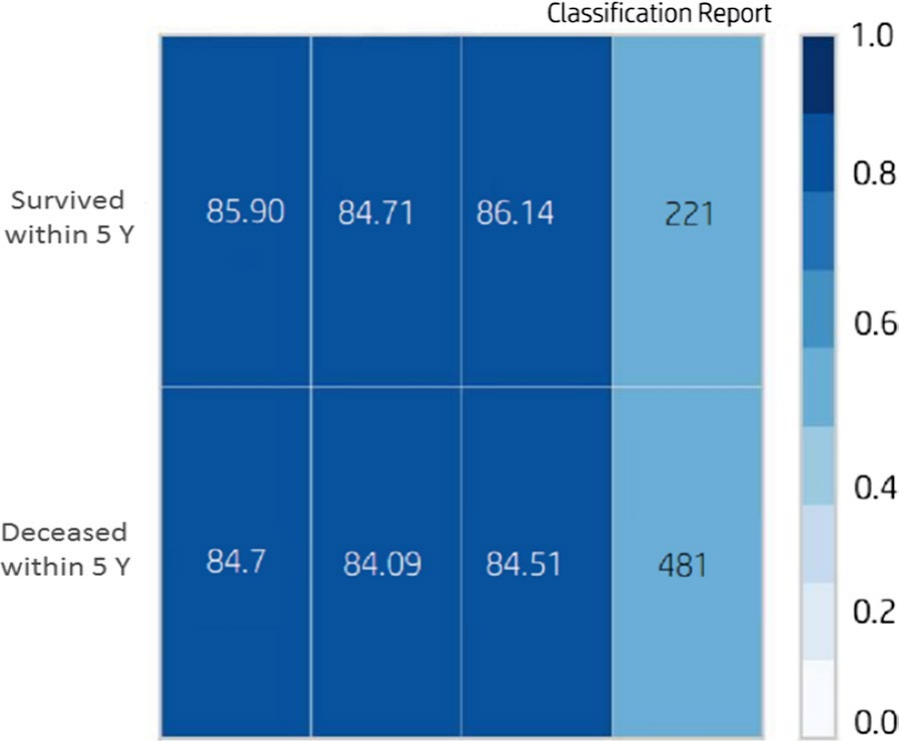
Classification report for SVM with RBF kernel

Based on Fig. 3a, b, the results obtained for SVM with RBF kernel on the selected features outperformed the other seven ML techniques, yielding 85.7% for average accuracy, 85% for specificity, 86% for sensitivity, 87% for F-measure metrics, 86.1% for mean kappa statistic, and 85% for mean AUC metrics. Based on the full features dataset, the decision tree algorithm obtained the highest performance for all the assessment criteria. The second-highest performance on the selected-features and full-features datasets belonged to J-48 and SVM with RBF kernel for the prediction of the 5-year CML survival. Finally, the worst result was obtained by MLP with a mean accuracy of 77%, a mean sensitivity of 72%, a mean specificity of 76%, a mean F-measure of 76%, a mean kappa statistic of 76.2%, and AUC of 76%. The AUC curve for the top eight ML algorithms and the classification report of the SVM with RBF kernel is depicted in Figs. 4 and 5, respectively.

## Discussion

This study retrospectively analyzed the data of 837 CML patients to develop an intelligent model for predicting the 5-year survival of CML patients. First, the most important variables affecting CML survival were identified using the mRMR feature selection technique. Then, the dataset with full and selected variables was fed into the ML models separately. Finally, the models’ performance was evaluated and compared based on the confusion matrix criteria. Initially, feature selection analysis was performed to select the most important variables. Among a total of 45 primary variables, a set of 12 variables (about 27%) including age, sex, spleen palpable, unexplained hemorrhage, increased leucocyte count, elevated blast cell proportion, resistance to a tyrosine kinase inhibitor, decreased platelet counts, anemia, malaise, and night sweats were selected as the most important predictors affecting CML. These variables are largely consistent with those in reviewed studies. The results of comparing the eight selected ML algorithms after feature selection showed that the SVM RBF achieved the highest performance in the 5-year survival prognosis of CML patients with an accuracy of 85.7%, specificity of 85%, sensitivity of 86%, F-measure of 87%, AUC of 0.85, and kappa statistic of 86.1%.

Feature selection is a key prerequisite for data mining, which reduces unnecessary data and improves the speed and efficiency of data mining [38]. Previous studies showed that numerous clinical and non-clinical predictors influence CML survival. In reviewed studies, after performing feature selection, a number of demographical and clinical manifestation variables such as age [27, 31, 39, 40], sex [14, 27, 40], body mass index (BMI) [10, 27, 30, 31], race [27, 40], body pain [14, 25, 27, 28, 40, 41], general malaise [10, 14, 27, 31, 40], fever [10, 26, 28–30], night sweats [25, 28–30, 41], unexplained hemorrhage [10, 26, 28, 30, 39], general infection [14, 31, 40], enlarged spleen [10, 14, 27, 31, 39, 40], cachexia [25, 27, 30, 31], anorexia [10, 14, 29, 31], and drug resistance [25, 27–29, 41] are determined as the most important predictors affecting CML survival. Besides, neutrophil/lymphocyte count [10, 14, 25–27, 29, 31, 39], lactate dehydrogenase (LDH) [14, 25–27, 29], complete blood count (CBC) [14, 25, 26, 28, 29], platelet count [10, 25, 26, 40, 41], peripheral blast count (PBC) [14, 25, 27, 29, 41], and red blood cell (RBC) count [14, 25–28, 39, 40] are considered as the paramount laboratory and evaluative variables of CML.

The results of our study are consistent with the studies by Eckardt et al.[28], Coombes et al. [41], and Orgueira et al. [14] In these studies, after implementing various ML models to predict the survival of patients with leukemia, the SVM classifier yielded the best performance. In a study by Karami et al., the SVM had better performance with 85.17% accuracy and 0.93% AUC for the survival prognosis of AML patients [30]. Taiwo et al. evaluated the performance of four ML algorithms to predict the survival of patients with CML. Their results showed that the SVM algorithm would present a better performance with 99.82% classification accuracy [10]. Furthermore, Chen et al. compared the performance of three ML methods for the survival prognosis of chronic lymphocytic leukemia (CLL) patients. Finally, the SVM model with 90% AUC exhibited the best performance [39].

Contrary to the results of the present study, in some studies such as Das et al. [27] and Hauser et al. [31], ensemble models performed better in predicting the survival of CML patients. Das et al. found that gradient boosting with an AUC of 0.77 could best help survival prognosis amongst the selected methods [27]. Hauser et al. also showed that ML technologies, in particular XGBoost and LASSO models, would help with active patient survival prognosis and prompt identification of high-risk CML cases for treatment improvement and care planning purposes (AUC range: 0.87–0.96) [31]. Hu et al. (2021) revealed that ML algorithms offered an effective predictive model for timely, effective, and economical identification and prognosis of leukemia and its survivability [40].

## Limitations and implications

The proposed model is likely to accurately predict the 5-year survival of patients with CML. Hence, this can make the designed model applicable to real clinical settings. However, the present study faced some potential limitations. First, as we used a retrospective dataset, there were certain missing and noisy fields (e.g., incoherent, incomplete, abnormal, meaningless, and erroneous) that could have impacted the modeling process. Therefore, to deal with noisy fields, the normal range of each variable was defined using the opinion of two oncologists. Then, we specified all the values that fell outside the defined range (noisy fields) and completed them by referring to patient records or the responsible physician. In addition, the records with more than 70% of empty fields were removed and imputed by mean or mode values for continuous and discrete variables, respectively. Second, we dealt with a single-center dataset with a limited sample size that undoubtedly affected the quality of modeling, comprehensiveness, and generalizability of data. Third, the dataset did not collect data on economic status, lifestyle habits, molecular biology, genomic, proteomic, or metabolomic factors that may affect the survival of CML patients. The inclusion of these factors may increase the predictive power of the models. Furthermore, the dynamic nature of some variables would double the need to use systematic follow-up programs for a more comprehensive picture of the patient. Therefore, it is recommended that more studies be conducted after more accurate validations to improve the quality of modeling and minimize prognosis bias.

## Conclusions

ML techniques as new, innovative, and non-invasive methods for the 5-year survival prognosis of CML patients will improve healthcare quality services, offer customized treatment, and reduce the serious complications and deaths associated with the disease. Therefore, we implemented and compared the performance of eight ML-based models for 5-year survival prediction of CML patients. After identifying the most important predictor variables (12 variables) and implementing the classification models, the SVM (RBF kernel) algorithm with an AUC of 0.856 presented the best performance. The algorithm proposed for effective identification of high-risk patients and predicting disease behavior and complications will effectively help medical experts to maintain treatment cost-effectiveness, prioritize resources, and improve safety and care quality. At the same time, it will improve patients' life expectancy. In future studies, our proposed models are expected to be customized to other malignancies and clinical areas. It is also recommended that more ML and even deep learning (DL) techniques be adopted for a profound and more reflective analysis, system user interface implementation, and system external validation in the real clinical environment. We expect our model to be further validated and probably re-optimized based on mixed datasets from multiple settings. While external validation is required, our developed model provides a basis to develop intelligent systems for CML disease.

## Data Availability

The datasets used and/or analyzed during the current study are available from the corresponding author on reasonable request.

## Abbreviations

CML: Chronic myeloid leukemia
ML: Machine learning
MLP: Multilayer perceptron
SVM: Support vector machine
mRMR: Minimal redundancy maximal relevance
CMD: Clonal myeloproliferative disorder
AAO: Mean age-at-onset
AI: Artificial intelligence
CRISP: Cross-industry standard process
EMR: Electronic medical record system
CI: Confidence interval
AUC: Area Under the Curve
AML: Acute myeloid leukemia
LDH: Lactate dehydrogenase
WBC: White blood cell
TNC: Total nucleated cell
BMI: Body mass index
CLL: Chronic lymphocytic leukemia
